# Gene Co-Expression Network Analysis Reveals the Hub Genes and Key Pathways Associated with Resistance to *Salmonella* Enteritidis Colonization in Chicken

**DOI:** 10.3390/ijms24054824

**Published:** 2023-03-02

**Authors:** Qiao Wang, Mamadou Thiam, Astrid Lissette Barreto Sánchez, Zixuan Wang, Jin Zhang, Qinghe Li, Jie Wen, Guiping Zhao

**Affiliations:** State Key Laboratory of Animal Nutrition, Institute of Animal Sciences, Chinese Academy of Agricultural Sciences, Beijing 100193, China

**Keywords:** chicken, transcript factors, *Salmonella*, cecal microbiome, SCFAs

## Abstract

*Salmonella* negatively impacts the poultry industry and threatens animals’ and humans’ health. The gastrointestinal microbiota and its metabolites can modulate the host’s physiology and immune system. Recent research demonstrated the role of commensal bacteria and short-chain fatty acids (SCFAs) in developing resistance to *Salmonella* infection and colonization. However, the complex interactions among chicken, *Salmonella*, host–microbiome, and microbial metabolites remain unelucidated. Therefore, this study aimed to explore these complex interactions by identifying the driver and hub genes highly correlated with factors that confer resistance to *Salmonella*. Differential gene expression (DEGs) and dynamic developmental genes (DDGs) analyses and weighted gene co-expression network analysis (WGCNA) were performed using transcriptome data from the cecum of *Salmonella* Enteritidis-infected chicken at 7 and 21 days after infection. Furthermore, we identified the driver and hub genes associated with important traits such as the heterophil/lymphocyte (H/L) ratio, body weight post-infection, bacterial load, propionate and valerate cecal contents, and *Firmicutes*, *Bacteroidetes*, and *Proteobacteria* cecal relative abundance. Among the multiple genes detected in this study, *EXFABP*, *S100A9/12*, *CEMIP*, *FKBP5*, *MAVS*, *FAM168B*, *HESX1*, *EMC6*, and others were found as potential candidate gene and transcript (co-) factors for resistance to *Salmonella* infection. In addition, we found that the PPAR and oxidative phosphorylation (OXPHOS) metabolic pathways were also involved in the host’s immune response/defense against *Salmonella* colonization at the earlier and later stage post-infection, respectively. This study provides a valuable resource of transcriptome profiles from chicken cecum at the earlier and later stage post-infection and mechanistic understanding of the complex interactions among chicken, *Salmonella*, host–microbiome, and associated metabolites.

## 1. Introduction

*Salmonella* infections threaten the poultry industry and public health. While spontaneous *Salmonella* spp. infection is unlikely to result in a considerable number of chicken deaths, it will have a significant detrimental impact on poultry production capacity and health. Additionally, it is a zoonotic disease that poses a significant hazard to public health and safety [1,2,3,4,5]. Therefore, it is crucial to understand the mechanisms of the complex interactions among chicken, *Salmonella*, and host–microbiome to minimize economic losses in poultry production and protect animal and human health [6].

The gut microbiota of chickens is diverse and complex, and it is critical for nutrition, immune system development, and pathogen exclusion. In their study, Kempf and co-authors defined super shedding as a shed of high levels of pathogens resulting from successful infection and colonization persistence in the ceca [7]. They also hypothesized that a high diversity or the presence of specific features of the gut microbiota inhibits pathogens growth [7]. The gut microbiota can protect against harmful bacteria by sticking to the intestinal epithelial walls [8]. These bacteria are capable of producing many compounds involved in the barrier effect such as short-chain fatty acids (SCFAs: acetate, propionate, and butyrate), organic acids (lactic acid), and antibacterial chemicals (bacteriocins), as well as generating non-pathogenic immunological responses that benefit the animal by providing sustenance and protection [8,9,10]. Commensal bacteria provide fundamental benefits to the host by providing nutrients, competitively excluding pathogens or non-native germs, and stimulating and training the immune system [11]. Furthermore, the host’s intestinal microbiota can promote the development of the immune system, which includes the intestinal epithelial cells, mucus layers, intestinal immune cells, and lamina propria [1,11,12].

Previous studies demonstrated that chickens and other avian species with low heterophil/lymphocyte (H/L) ratio are more resistant to environmental stressors than birds with a high H/L ratio [13,14]. Recent studies have suggested that a low H/L ratio provides resistance benefits such as better immune response, performance, adaptability, and longevity [15,16]. Our previous study demonstrated that chickens with low H/L are more resistant, which could be associated with increased cecal relative abundance in *Proteobacteria* and *Bacteroidetes* [17], thus suggesting that the commensal *Proteobacteria* and *Bacteroidetes* could be involved in this resistance against *Salmonella* through diverse mechanisms not well understood. To date, no study has been conducted to examine the complex interactions between chicken, *Salmonella*, and host–microbiome by assessing gene regulation and correlation with the resistance to *Salmonella* infection. Therefore, with the aim to contribute to developing better understanding of these mechanisms, the current study was initiated to identify the candidate genes and signaling pathways associated with the resistance to *Salmonella* mediated by the intestinal microbiota and derived metabolites. Through this, we have identified the differentially expressed, developmentally dynamic driver and hub genes associated with the resistance to *Salmonella* and correlated with factors such as the body weight post-infection, H/L ratio, bacterial load, propionate and valerate cecal contents, and *Firmicutes*, *Bacteroidetes*, and *Proteobacteria* cecal relative abundance by combining data from the transcriptome and weighted gene co-expression network analysis (WGCNA) of the cecum from chickens infected with *Salmonella* Enteritidis 7 and 21 days after infection. This may be valuable for the understanding of the mechanisms of resistance to *Salmonella* infection.

## 2. Results

### 2.1. Phenotypic, Immune, and Microbiome Diversity

**Figure 1:**
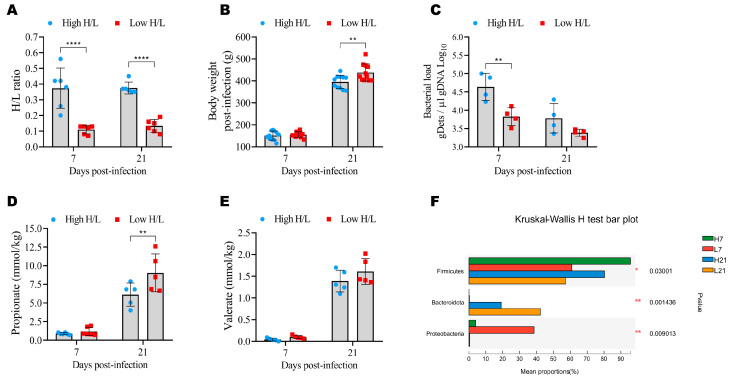
Phenotypic, cecal microbiota and metabolite differences. (**A**) Heterophil/lymphocyte (H/L) ratio of different groups (n = 6). (**B**) Body weight differences between low and high H/L ratio groups at 7 and 21 dpi (n = 11). (**C**) Bacterial load (from the cecum) differences between low and high H/L ratio groups at 7 and 21 dpi (n = 4). (**D**) Propionate differences between low and high H/L ratio groups at 7 and 21 dpi (n = 5). (**E**) Valerate differences between low and high H/L ratio groups at 7 and 21 dpi (n = 5). (**F**) Cecal microbiome relative abundance (at the phylum level) differences between low and high H/L ratio groups at 7 and 21 dpi. Data were analyzed by the Kruskal–Wallis H test, with reported *p*-value and significance. H7: High H/L SE-infected 7 dpi (n = 8); L7: Low H/L SE-infected 7 dpi (n = 7); H21: high H/L SE-infected 21 dpi (n = 6); L21: Low H/L SE-infected 21 dpi (n = 7). * *p* < 0.05, ** *p* < 0.01, **** *p* < 0.0001. Data analysis was performed using 2-way ANOVA with Sidak’s multiple comparisons, with alpha = 0.05.

To evaluate the resistance to *Salmonella* infection mediated by the gut microbiota and derived metabolites between high and low H/L ratio *Salmonella* Enteritidis (SE)-infected chickens, we measured the H/L ratio (at 7 days old), body weight post-infection (BW), bacterial load, propionate and valerate contents, and the cecal microbiota relative abundance at 7 and 21 days post-infection (dpi). The H/L ratios of chickens with high and low H/L ratio were significantly (*p* < 0.0001) different at 7 and 21 dpi (Figure 1A). It was noteworthy that chickens with a low H/L ratio showed significantly reduced body weight loss compared to chickens with a high H/L ratio at 21 dpi (Figure 1B). The determination of SE load in the cecum tissues revealed that the chickens with low H/L ratio displayed a lower bacterial load than chickens with a high H/L ratio, with a significant difference (*p* = 0.0053) observed at 7 dpi (Figure 1C). Regarding propionate and valerate cecal contents, we observed that the chickens with a low H/L ratio were characterized by increased concentration of these microbial metabolites (Figure 1D,E). To assess the cecal microbiome composition of chickens with high and low H/L ratios, 16S rRNA sequencing analysis was performed at 7 and 21 dpi. In this study, the more abundant phyla were *Firmicutes* (*p* = 0.030), *Bacteroidetes* (*p* = 0.0014), and *Proteobacteria* (*p* = 0.0090), with a differential abundance among groups over time points post-infection (Figure 1F).

### 2.2. Transcriptome Profiling and Differentially Expressed Genes (DEGs)

**Figure 2:**
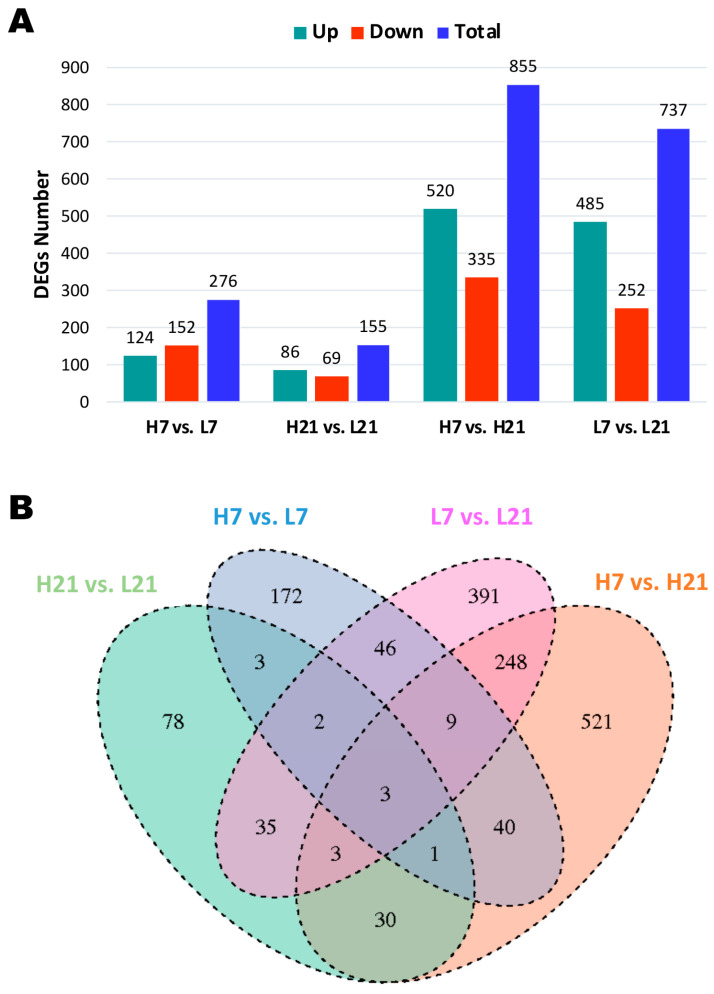
Identification of differentially expressed genes (DEGs) in low and high H/L ratio chicken groups at 7 and 21 dpi. (**A**) Summary of total DEGs between low and high H/L ratio groups. (**B**) Venn diagram showing the number of unique and shared DEGs between different groups. H7: High H/L SE-infected 7 dpi (n = 6); L7: Low H/L SE-infected 7 dpi (n = 7); H21: high H/L SE-infected 21 dpi (n = 5); L21: Low H/L SE-infected 21 dpi (n = 4).

The number of DEGs detected between high and low H/L ratio SE-infected chickens at 7 (H7 and L7), and 21 (H21 and L21) dpi varied from 155 to 855 (Figure 2A). To obtain an insight into the cecum gene transcription among the four groups, we performed the following comparison: H7 vs. L7, H21 vs. L21, H7 vs. H21, and L7 vs. L21. The results showed more DEGs in the comparison between same H/L ratios level (high or low) at 7 and 21 dpi (H7 vs. H21 with 855 DEGs and L7 vs. L21 with 737 DEGs; Figure 2A). However, a low number of DEGs was observed in comparing high and low H/L ratio chickens at 7 or 21 dpi (H7 vs. L7 with 276 DEGs and H21 vs. L21 with 155 DEGs; Figure 2A). The overlapping genes among these four features of comparison are shown in Figure 2B.

The top DEGs identified from the different comparisons are shown in Table 1. From these comparisons, four potential genes were identified as involved in the defense against *Salmonella* infection. Immune-related gene such as *FKBP5* (H7 vs. L7) was upregulated in chickens with a low H/L ratio than in chicken with a high H/L ratio at 7 dpi. Compared to chicken with a high H/L ratio, chicken with a low H/L ratio showed upregulation of *CEMIP* gene expression at 21 dpi (H21 vs. L21). Moreover, we detected that *EXFABP* and *S100A9* genes were significantly and differentially expressed between 7 and 21 dpi in chickens with low H/L ratios (L7 vs. L21). They were upregulated at 7 dpi in comparison to 21 dpi.

**Figure 3:**
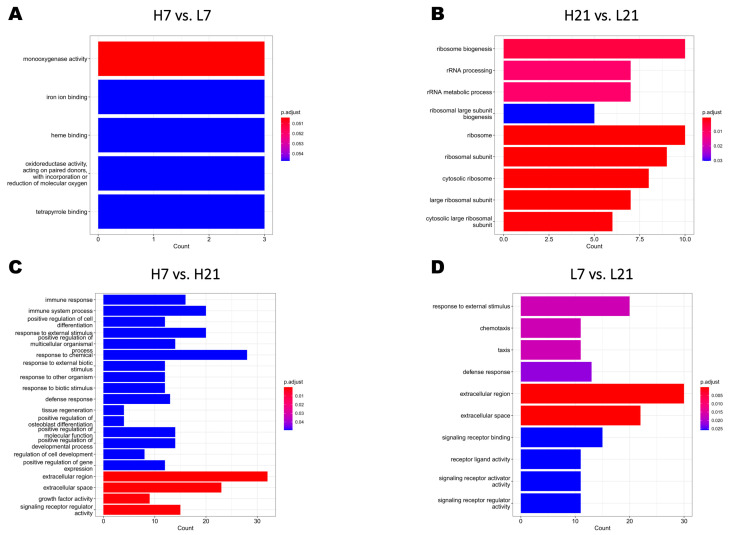
Identification of Gene Ontology (GO) terms of different groups. (**A**) H7 vs. L7 GO terms. (**B**) H21 vs. L21 GO terms. (**C**) H7 vs. H21 GO terms. (**D**) L7 vs. L21 GO terms. H7: High H/L SE-infected 7 dpi (n = 6); L7: Low H/L SE-infected 7 dpi (n = 7); H21: high H/L SE-infected 21 dpi (n = 5); L21: Low H/L SE-infected 21 dpi (n = 4).

**Figure 4:**
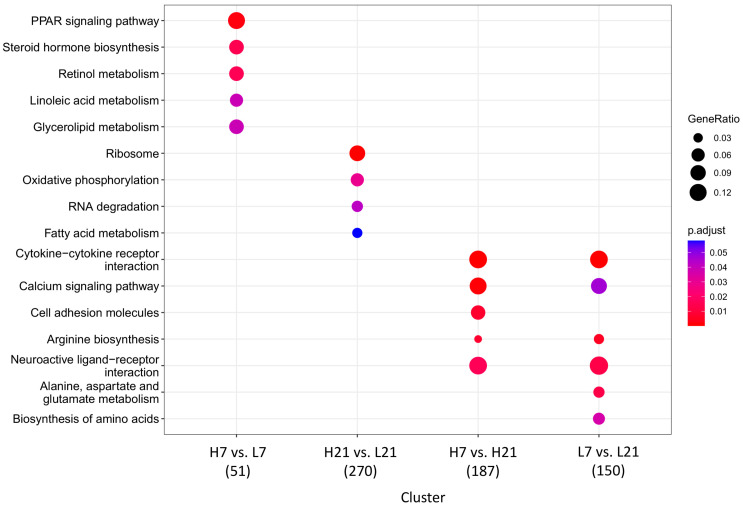
Kyoto Encyclopedia of Genes and Genomes (KEGG) pathways enrichment of different groups. H7: High H/L SE-infected 7 dpi (n = 6); L7: Low H/L SE-infected 7 dpi (n = 7); H21: high H/L SE-infected 21 dpi (n = 5); L21: Low H/L SE-infected 21 dpi (n = 4).

To identify the biological process regulated by the significant DEGs and their effect on the resistance to *Salmonella* infection, Gene Ontology (GO) enrichment (Figure 3) and Kyoto Encyclopedia of Genes and Genomes (KEGG) pathways (Figure 4) analyses were performed for each of the four versus. The results showed that the four versus displayed different numbers and categories. Biological processes such as monooxygenase activity and ribosome-related regulation were significantly enriched in H7 vs. L7 (5 GO terms) and H21 vs. L21 (9 GO terms), respectively (Figure 3A,B). It was noteworthy that only the versus comparing the same H/L ratio level between the two-time points post-infection showed immune-related GO (Figure 3C,D). The KEGG pathways analysis of detected DEGs from the four versus is presented in Figure 4. The PPAR signaling pathway and oxidative phosphorylation were significantly enriched in H7 vs. L7 and H21 vs. L21, respectively (Figure 4). Pathways such as cytokine−cytokine receptor interaction and calcium signaling pathway were significantly enriched and both detected between H7 vs. H21 and L7 vs. L21 (Figure 4). The genes such as *EXFABP* and *S100A12* were detected as involved in the GO terms immune response, response to external stimulus, response to other organisms, defense response, and extracellular region, which were enriched in H7 vs. H21 and L7 vs. L21. The detailed GO terms enrichment and KEGG pathways of the four versus are shown in Supplementary Table S2.

### 2.3. Developmental Dynamics Genes and Gene Expression Patterns in the Cecum

To explore the gene expression changes during *Salmonella* infection in the cecum, the genes with significant temporal changes (DDGs) were detected. Between the two times post-infection tested (7 and 21 dpi), 1290 genes were identified as DDGs, including 86 Transcript Factors (TFs) and 79 Transcription Co-Factors (TCFs) (Supplementary Table S3). Based on the DDGs analysis, 877 and 886 genes were identified as significant DDGs in high and low H/L ratio SE-infected chicken groups, respectively. The Venn analysis showed with 842 shared DDGs between high (35 unique DDGs) and low (44 unique DDGs) H/L ratio chickens (Supplementary Figure S1A). The top 20 enriched GO terms for these TFs of DDGs are shown in Supplementary Figure S1. From the GO analysis, genes such as *HESX1* and *SMAD5* were involved in most biological processes and identified as potential immune-related DDGs. The KEGG analysis for these TFs of DDGs showed four enriched KEGG pathways, including C-type lectin receptor signaling pathway, influenza A, adipocytokine signaling pathway, and TGF-beta signaling pathway (Supplementary Figure S1C). Genes such as *IRF1*, *RAF1*, *NFATC3*, *NFKBIB*, and *JAK2* were involved in these KEGG pathways and identified as key immune DDGs. Detailed GO and KEGG information for the TFs are shown in Supplementary Table S4.

### 2.4. Weighted Gene Co-Expression Network Analysis (WGCNA)

**Figure 5:**
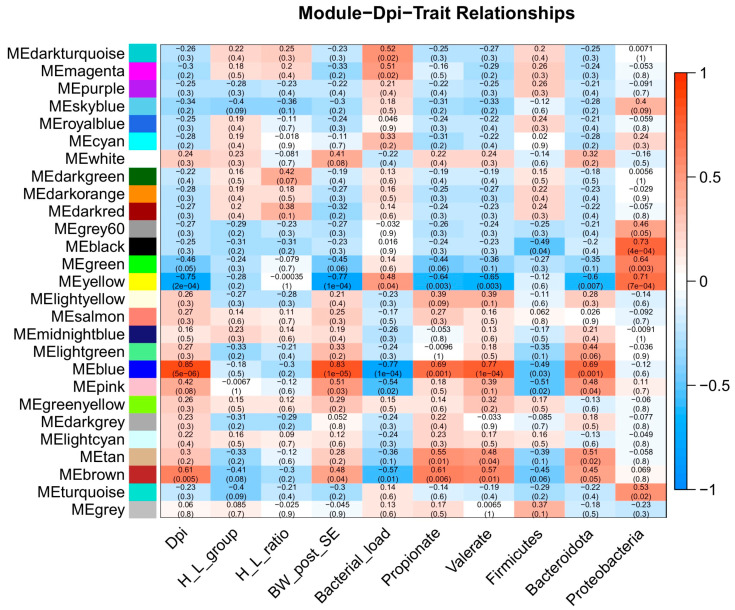
Heat map of module-trait relationships, each cell has two values. The upper is the absolute value of the correlation coefficient, and the down is the *p*-value. Red and blue colors represent positive and negative correlations, respectively. Dpi: day post-infection; H_L: H/L ratio; BW_post_SE: body weight after *Salmonella* Enteritidis infection.

Weighted Gene-Co-Expression Network Analysis (WGCNA) was performed using factors of interest such as days post-infection (dpi), H/L ratio, body weight post-infection (BW), bacterial load, propionate and valerate cecal contents, and *Firmicutes*, *Bacteroidetes*, and *Proteobacteria* cecal relative abundance (Supplementary Figure S2A). In the present study, cecum transcriptome data were used to construct the expression matrix, and traits data from high and low H/L ratio SE-infected chickens at 7 and 21 dpi were combined and analyzed (including 19 samples). A total of 19,501 genes were obtained to build the weighted gene co-expression network after removing the offending genes. First, we determined the best soft threshold (8) using the scale-free topological model and mean connectivity (Supplementary Figure S2B). Next, the cluster dendrogram of co-expression network modules was generated using hierarchical clustering of genes based on the 1-TOM formula (Supplementary Figure S2C). As a result, 27 co-expression modules were obtained, and the corresponding modules–traits relationships are presented in Figure 5.

**Figure 6:**
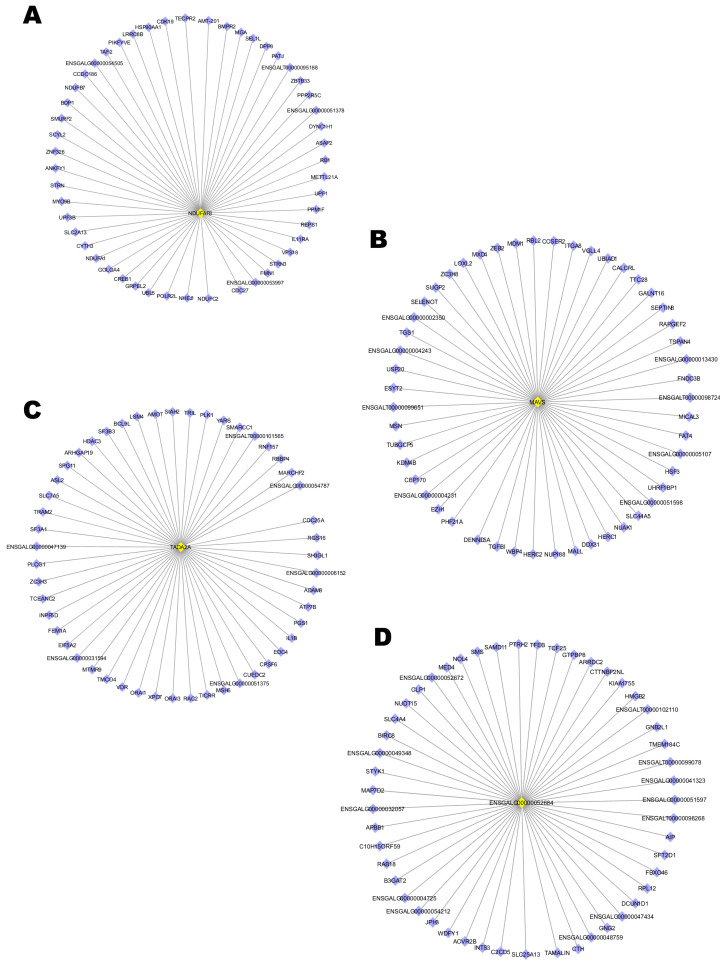
Co-expression network of hub genes. (**A**) Hub genes of the blue module. (**B**) Hub genes of the brown module. (**C**) Hub genes of the green module. (**D**) Hub genes of the yellow module.

Ten modules were identified as highly correlated with the factors, including dark-turquoise, magenta, dark-green, black, green, yellow, blue, pink, tan, and brown (Figure 6). It was noteworthy that the yellow module was significantly and negatively correlated with the majority of factors, while the blue module was significantly and positively correlated with the factors (Figure 5). The blue (r = 0.85, *p* = 5e−06) and brown (r = 0.61, *p* = 0.005) modules were significantly and positively correlated with dpi, while the yellow module (r = −0.75, *p* = 2e−04) was significantly and negatively correlated (Figure 5). Based on the results obtained no modules were significantly correlated with H/L ratio. However, the dark-green module was positively correlated with H/L ratio (r = 0.42, *p* = 0.07; Figure 5). The yellow module (r = −0.77, *p* = 1e−04) was significantly and negatively correlated with body weight post-infection, while the blue module (r = 0.83, *p* = 1e−05) was significantly and positively correlated (Figure 5). The dark-turquoise (r = 0.52, *p* = 0.02) and magenta (r = 0.51, *p* = 0.02) modules were significantly and positively correlated with bacterial load, while the blue (r = −0.77, *p* = 1e−04) and the brown (r = −0.57, *p* = 0.01) modules were significantly and negatively correlated. Concerning the microbial metabolites, the yellow module was significantly and negatively correlated with propionate and valerate (r = −0.64, *p* = 0.003 and r = −0.65, *p* = 0.003, respectively), while the blue (r = 0.69, *p* = 0.001 and r = 0.77, *p* = 1e−04, respectively), tan (r = 0.55, *p* = 0.01 and r = 0.48, *p* = 0.04, respectively), and the brown (r = 0.61, *p* = 0.006 and r = 0.57, *p* = 0.01, respectively) modules were significantly and positively correlated (Figure 5). The black (r = −0.49, *p* = 0.04), blue (r = −0.49, *p* = 0.03), and pink (r = −0.51, *p* = 0.02) modules were significantly and negatively correlated with *Firmicutes* relative abundance (Figure 5). The *Bacteroidetes* relative abundance was significantly and negatively correlated with the yellow module (r = −0.60, *p* = 0.007), while significantly and positively correlated with blue module (r = 0.69, *p* = 0.001; Figure 5). It was remarkable that no modules were significantly and negatively correlated with *Proteobacteria* relative abundance, whereas the black (r = 0.73, *p* = 4e−04), green (r = 0.64, *p* = 0.003), and yellow (r = 0.71, *p* = 7e−04) modules were significantly and positively correlated with *Proteobacteria* relative abundance (Figure 5).

In addition, the top 10 driver genes of the interesting modules were identified according to the absolute value of gene significance (|GS| > 0.5) and module membership (|MM| > 0.5). As a result, the top 10 driver genes of the interesting module are shown in Table 2.

### 2.5. Screening of Hub Genes Associated with Resistance to Salmonella

To identify the hub genes involved in the resistance to *Salmonella* in chickens’ cecum, we selected 4 key modules (blue, brown, green, and yellow) strongly correlated with the factors. The co-expression network with detected hub genes of the key modules selected are shown in Figure 6. The genes such as *NDUFAF8*, *MAVS*, *TADA2A*, and *ENSGALG00000052684* identified in the top DEGs and driver genes were identified as hub genes in the blue (Figure 6A), brown (Figure 6B), green (Figure 6C), and yellow module (Figure 6D), respectively.

**Figure 7:**
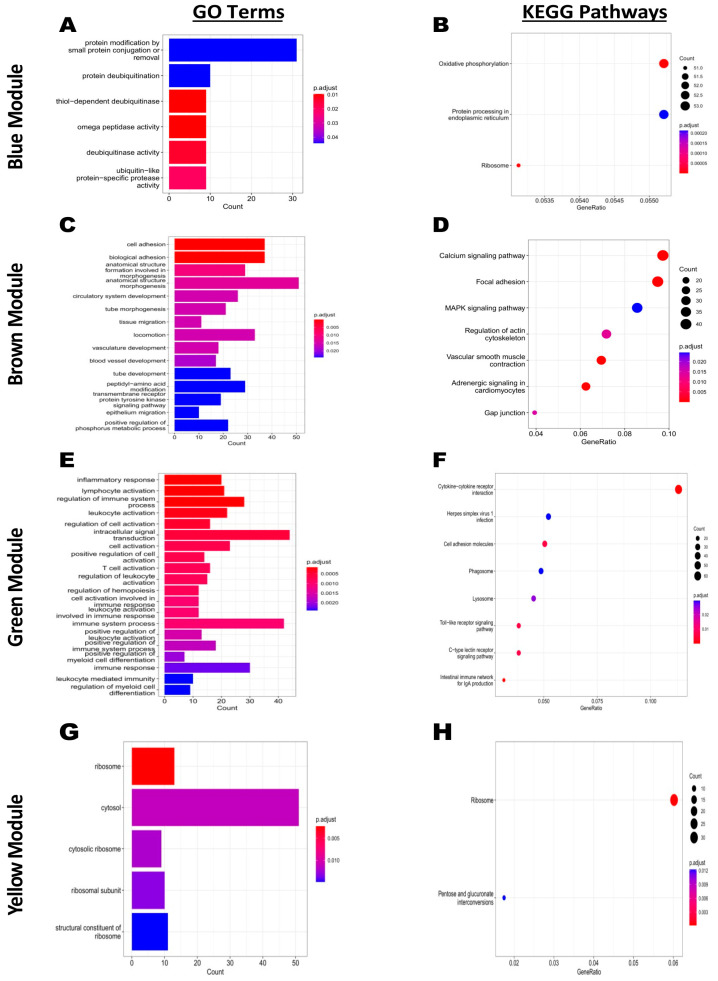
GO terms and KEGG pathways enrichment of interesting modules. (**A**,**B**) GO and KEGG of genes contained in the blue module, respectively. (**C**,**D**) GO and KEGG of genes contained in the brown module, respectively. (**E**,**F**) GO and KEGG of genes contained in the green module, respectively. (**G**,**H**) GO and KEGG of genes contained in the yellow module, respectively.

To assess the biological process regulated by the hub genes and their effect on the resistance to *Salmonella* infection, GO enrichment and KEGG pathways analyses were performed for the genes detected in the blue, brown, green, and yellow modules (Figure 7). The KEGG analysis showed that the oxidative phosphorylation metabolic pathway was significantly enriched by the cluster of genes from the blue module. It was noteworthy that among the four modules selected, the green module showed more enriched GO terms and KEGG pathways related to the immunity than the blue, brown, and yellow modules (Figure 7). Among the top 20 GO terms significantly enriched in the green module, inflammatory response, lymphocyte activation, regulation of immune system, T cell activation, immune response, leukocyte activation, positive regulation of myeloid cell differentiation, and leukocyte-mediated immunity were significantly enriched (Figure 7E); eight KEGG pathways were significantly enriched in this module, cytokine−cytokine receptor interaction, herpes simplex virus 1 infection, cell adhesion molecules, phagosome, lysosome, Toll-like receptor signaling pathway, C-type lectin receptor signaling pathway, and intestinal immune network for IgA production (Figure 7F). The detailed information on GO enrichment and KEEG pathways analyses of the four interesting modules are shown in Supplementary Table S5.

### 2.6. Identification of Major Driver Genes by the Overlapping Method

To identify the driver genes from the significant module–traits relationship, the genes were screened according to their gene significance (GS), module membership (MM), and *p*-value (*p* < 0.01). The top 10 driver genes of the interesting module–traits relationship are presented in Table 2.

**Figure 8:**
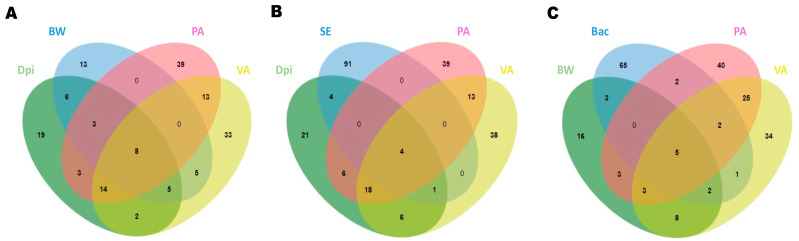
Overlap of driver gene contained in all the modules identified as highly correlated with the indicated trait. (**A**) Dpi (day post-infection) vs. BW (body weight post-infection) vs. PA (propionate cecal content) vs. VA (valerate cecal content). (**B**) Dpi (day post-infection) vs. SE (bacterial load from cecum) vs. PA (propionate cecal content) vs. VA (valerate cecal content). (**C**) BW (body weight post-infection) vs. Bac (Bacteroides cecal relative abundance) vs. PA (propionate cecal content) vs. VA (valerate cecal content).

To detect the genes significantly correlated with some interesting factors, a Venn diagram analysis was performed to identify the genes shared between four factors that have been demonstrated to play a role in the resistance to *Salmonella* infection (Figure 8). The *FAM168B*, *RAF1*, *HESX1*, *USP8*, *C2CD5*, *PIGC*, *ENSGALG00000053041*, and *ENSGALT00000092369* were found to be top driver genes shared among dpi, body weight post-infection, and propionate and valerate cecal contents (Figure 8A). Concerning the genes shared in the top driver from dpi, bacterial load, and propionate and valerate cecal contents, *EMC6*, *NFU1*, *ENSGALG00000021686*, and *ENSGALG00000048205* were identified (Figure 8B). The gut microbiota can modulate the immune system of the host through SCFAs or direct inhibition, and it is in this optic that we identified *FAM168B*, *RAF1*, *HESX1*, *PIGC*, and *ENSGALT00000092369* as the top driver genes shared among body weight post-infection, *Bacteroidetes* relative abundance, and propionate and valerate cecal contents (Figure 8C). The detailed information of unique and shared genes among multiple combinations of four factors is shown in Supplementary Table S6.

### 2.7. Verification of Selected Candidate Genes Involvement in the Process of Salmonella Infection in Chicken

**Figure 9:**
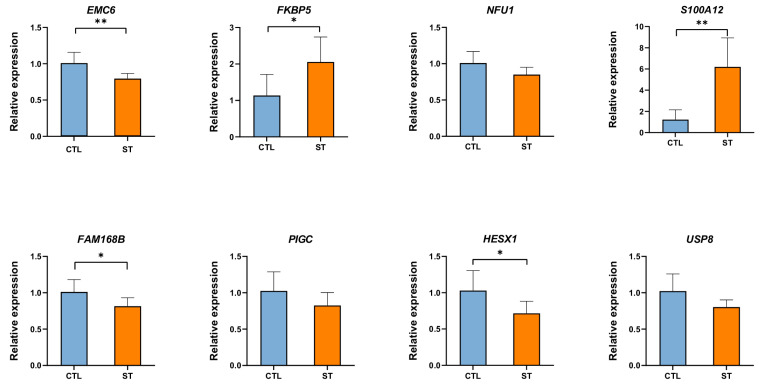
Expression levels of selected candidate genes from cecum tissues of Dagu chickens non-infected and ST-infected 24 h post-infection. CTL refers to the chickens from the non-infected group, while ST corresponds to the ST-infected chickens. Six birds were used per group. Data were analyzed using Student’s *t*-test. * *p* < 0.05, ** *p* < 0.01.

To verify the implication of some candidate genes in the process and acquisition of resistance to *Salmonella* infection in chicken, we quantified the expression of *EMC6*, *FKBP5*, *NFU1*, *S100A12*, *FAM168B*, *PIGC*, *HESX1*, and *USP8* in cecum tissues of non-infected and *Salmonella* Typhimurium (ST)-infected Dagu chickens using qRT-PCR. Through this, we were able to assess whether the expression level of these genes is associated to the resistance to pathogenic infections such as *Salmonella*. Figure 9 shows that compared to the control group, the gene expression of *FKBP5* and *S100A12* increased significantly after *Salmonella* infection, while the expression of *EMC6*, *FAM168B*, and *HESX1* decreased significantly. The expression level of *NFU1*, *PIGC*, and *USP8* decreased under *Salmonella* infection, without a significant difference between non-infected and ST-infected chickens (Figure 9).

## 3. Discussion

Chickens with a low H/L ratio are superior to the chickens with a high H/L ratio in terms of survival, immune response, and resistance to *Salmonella* infection [13,14,15,18,19]. Our previous experiment demonstrated that the H/L ratio was linked to important features such as intestinal immunity, the inflammatory response, and the cecal microbiota composition in SE-infected chicken. In the present study, we performed a time course (at 7 and 21 dpi) transcriptome profiling of cecum tissues during SE infection to identify genes associated with important immune traits involved in *Salmonella* resistance directly or mediated by the gut microbiota and its metabolites. Therefore, this study provides valuable genetic resources on the mechanisms of resistance to *Salmonella* colonization in chickens.

*Salmonella* infections in poultry have been linked to reduced performances, intestinal colonization, inflammation, and deep organ invasion [20]. In the present study, under *Salmonella* infection, chickens with a low H/L ratio displayed increased body weight (at 21 dpi), propionate (at 21 dpi), valerate (at 21 dpi), and significantly higher *Proteobacteria* and *Bacteroidetes* cecal relative abundance at 7 and 21 dpi, respectively. Our previous study discussed these results, where we demonstrated that the H/L ratio modulates the cecal microbiota, and this modulation could be one of the multiple mechanisms of resistance to *Salmonella* infection [17]. Recent studies reported that chickens with a low H/L ratio were more resistant to *Salmonella* through increased IL-1β and IFN-γ blood serum concentration and intestinal expression and potentially through a particular cecal microbiota composition and SCFAs cecal content at specific days after infection [16,17,19]. Chickens acquire resistance to *Salmonella* infection with age due to the development of their gastrointestinal and immune systems [21,22]. The intestinal epithelial cells and mucus layers act as barriers between the host and the microbes, defending the host against undesirable gut microorganisms. Microbial metabolites can also modulate the immune system by affecting host cells’ physiology and gene expression [23,24,25]. The SCFAs possess bacteriostatic properties that inhibit the growth of foodborne pathogens such as *Salmonella* spp. [26].

The cecum transcriptome profiling performed in this study identified genes such as *FBXO32* (H7 vs. L7), *FKBP5* (H7 vs. L7), *NDUFAF8* and *CEMIP* (H21 vs. L21), *TIMD4* (H7 vs. H21), *PER2* (H7 vs. H21), *EXFABP* (L7 vs. L21), *MST1* (L7 vs. L21), and *S100A9* (L7 vs. L21) as candidate genes for resistance to *Salmonella* infection. The role of these genes in chicken is not clearly defined. Therefore, further investigations are needed to explain their function and possible involvement in chicken’s disease resistance. Among the genes detected, *FKBP5*, *CEMIP*, *EXFABP*, and *S100A9/12* are promising candidate genes for studying the mechanisms of resistance to *Salmonella* colonization. Although it is well established that FK506-binding protein 5 (FKBP5), a protein cochaperone, is involved with the inflammatory response, the regulatory mechanisms underlying leukocyte *FKBP5* DNA methylation remain unknown [27]. It has been reported that epigenetic *FKBP5* overexpression, a stress-induced protein cochaperone, is related to nuclear factor-B (NF-B)-mediated inflammation [28]. This gene has been linked to the control of NF-κB and IL-1.

In the current work, we detected that the peroxisome proliferator-activated receptors (PPARs) metabolic pathway was significantly enriched by the cluster of genes differentially expressed between chickens with low and high H/L ratios at 7 dpi. The PPARs control numerous pathways, such as modulation of the immune system and inflammatory response and the sensing of nutrients (fatty acids and their derivatives) [29]. Out of the three PPAR isotypes, PPARα can strongly inhibit inflammation through the repression of nuclear factor kappa B (NF-κB), activation of protein 1 (AP-1), as well as the signal transducer and activator of transcription (STAT) signaling pathways [29], whereas PPARγ is described as a double-edged sword, showing both pro- and anti-inflammatory effects and exerting beneficial as well as harmful effects upon host defenses against pathogenic bacteria [30]. PPARγ possesses anti-inflammatory effects via inhibition of pro-inflammatory molecules such as IL-6, TNF-α, IL-1β, and IL-12 [30]. The host and commensal bacteria can trigger PPARγ. In this context, Kelly and co-authors demonstrated that the commensal *Bacteroidetes Thetaiotaomicron* blocks the dysfunctional acute inflammatory response to infection by pathogenic *Salmonella* enterica by inducing binding of PPARγ to NF-κB RelA subunit and their joint nuclear export and cytosolic localization, resulting in the inhibition of the transcription of pro-inflammatory cytokine IL-8 [31]. Moreover, Grabacka and co-authors reported that microbiota products could influence PPARα signaling and, on the other hand, PPARα activation can affect microbiota profile, viability, and diversity [29]. It has been reported that PPARα activity is critical for maintenance of the intestinal barrier and the development of tolerance towards gut microbiota through suppression of Th1/Th17 inflammatory response [32]. PPARα-mediated IL-22 production by innate lymphoid cells has been described to be necessary for maintaining gut commensal microbiota homeostasis, protecting from pathogens, supporting beneficial microbiota, and suppressing unnecessary inflammation [29]. IL-22 is an IL-10 family cytokine, which is indispensable for the production of antimicrobial peptides such as regenerating islet-derived proteins RegIIIβ, RegIIIγ, calprotectin (S100A, S100B), as well as tight junction protein claudin 2; all these proteins are crucial to the host for control and clearance of intestinal pathogens [33,34]. Accordingly, in this study, *S100A9* was significantly and differentially expressed between low H/L ratio chicken 7 and 21 dpi (L7 vs. L21). *S100A9* was upregulated at 7 dpi in chicken with low H/L ratio (L7), compared to chicken with low H/L ratio at 21 dpi (L21). Moreover, the gene *S100A12* was involved in major immune-related GO terms identified across time points post-infection (H7 vs. H21 and L7 vs. L21). It is possible to suggest that the PPAR metabolic pathway and *S100A9/12* are involved in the resistance to *Salmonella* infection in chicken through unknown mechanisms.

The oleoylethanolamide (OEA) is an endogenously produced PPARα ligand (Grabacka et al., 2022) [29]. Recently, a study by Paola and co-authors demonstrated that administration of exogenous OEA to mice could increase microbial diversity and shift in colonic microbiota composition towards higher *Bacteroidetes* and lower *Firmicutes* abundance 11 days after inoculation [35]. Consistent with our results, we previously found that chickens with a low H/L ratio showed increased *Bacteroidetes* compared to chickens with high H/L ratio at 21 dpi [17]. We could hypothesize that PPAR pathway enrichment associated with increased expression of *S100A9/12* at 7 dpi is involved in the increased *Bacteroidetes* and resistance to *Salmonella* infection in chicken. The increased *Bacteroidetes* abundance was significantly and positively correlated with propionate and valerate cecal concentration [17]. In line with our hypothesis, a study performed on mice with high-fat diet (HFD)-induced diabetes revealed that mice treated with fenofibrate (synthetic PPARα agonist) had increased concentration of SCFAs (acetate, propionate, butyrate) [36]. These authors also reported that fenofibrate improved barrier functions of intestinal mucosa in HFD mice, visible by lower permeability and higher expression of genes encoding for tight junction proteins, zonula occludens 1 (ZO-1), and occluding in the colon [36]. Moreover, they also observed that the percentage of *Proteobacteria* group was also decreased after administration of the synthetic PPARα agonist, fenofibrate [36]. These reports and our findings suggest that the PPAR metabolic pathway could be involved in the shift of gut microbiota composition and the inhibition of *Salmonella* growth, respectively through an increase in *Bacteroidetes* and the antibacterial effect of the calprotectin *S100A*.

The gene *S100A12*, also known as calgranulin C [37], is a calcium-binding protein of the S100 subfamily of myeloid-related proteins that acts as an alarming signal to induce a pro-inflammatory innate immune response [38]. Yang and co-authors, in their study, reported that *S100A12* gene expression was very sensitive to low levels of LPS, indicating that exposure to higher levels of LPS enhances *S100A12* expression [39]. Interestingly, Hasegawa et al. reported that a PPAR-γ agonist inhibits *S100A12* expression by macrophages [40]. In accordance with our results, we found that PPAR signaling pathway was significantly enriched by the genes differentially expressed between chickens with low and high H/L ratios at 7 dpi. This result suggests that after induction of strong inflammatory response, the overexpression of PPAR was necessary for inhibition of inflammatory reactions.

Realegeno et al. [41], in their study, demonstrated that *S100A12* is involved in the antimicrobial network against *Mycobacterium leprae* in human macrophages. An important pathway for macrophage activation in innate immunity is through the recognition of bacterial lipoproteins by Toll-like receptor 2/1 heterodimers (TLR2/1) [42], which stimulates an antimicrobial response [43]. Realegeno et al. reported that *S100A12* is sufficient to directly kill *Mycobacterium tuberculosis* and *Mycobacterium leprae* and that is also required for TLR2/1L and IFN-γ induced antimicrobial activity against *M. leprae* in infected macrophages [41]. These observations, following our findings, suggest that *S100A12* plays a key role in macrophages’ antimicrobial activity via innate and adaptative immune response. However, to our knowledge, there is a lack of information regarding the role of *S100A12*-mediated antimicrobial activity against bacterial pathogens such *Salmonella* in macrophages. Interestingly, Komadath and co-authors, in a *Salmonella*-infected pig model and gene co-expression network analysis, identified *S100A12* among other genes as correlated with *Salmonella* shedding level and response to bacterial or *Salmonella* infection [44]. In line with this observation, Realegeno et al., in their study, identified *S100A12* in a module that was found to be significantly and positively correlated with TLR2/1L and associated with the Gene Ontology terms such as defense response, killing of cells of other organisms, chemotaxis, cytokine, and inflammatory response [41]. These observations constitute evidence that *S100A12* plays a key role in the host defense against pathogenic infection.

In this work, we found that the Oxidative phosphorylation (OXPHOS) pathway was significantly enriched in the cluster of genes differentially expressed between high and low H/L ratios chicken at 21 dpi. OXPHOS and mitochondrial reactive oxygen species (mtROS) are involved in multiple immune cell functions [45]. In addition, mitochondria are powerful organelles that can provide immunogenetic molecules, such as mitochondrial DNA (mtDNA), which triggers innate immune system activation [45]. M2 macrophage-mediated tissue repair and release of anti-inflammatory cytokine IL-10 often depends on the energy produced by OXPHOS and fatty acid oxidation [46]. Mitochondria also play a key role in NOD-like receptor family pyrin domain 3 (NLRP3) inflammasome activation [45]. Interestingly, in this study, we detected the gene co-expressed network of mitochondrial antiviral signaling protein (*MAVS*) as hub gene in the brown module. This module was significantly and positively correlated to propionate and valerate cecal concentration and *Bacteroidetes* relative abundance, while it was significantly and negatively correlated to *Salmonella* load. These results suggest that the OXPHOS pathway is involved in the control (inhibition) of *Salmonella* colonization through mechanisms involving *MAVS*. Accordingly, Park and co-authors, in their study, reported that *MAVS* regulates the production of type I IFN associated with NLRP3 [47]. The recruitment of NLRP3 leads to caspase-1-dependent secretion of pro-inflammatory cytokines, such as interleukin-1b (IL-1b) and IL-18 [45]. In line with our hypothesis that the OXPHOS pathway is associated with the inhibition of *Salmonella* growth through *MAVS*-mediated NLRP3 inflammasome activation, we reported in our previous study that a low H/L ratio is correlated with increased IL-1β and IFN-γ at 21 dpi [16]. In their study, Li and collaborators reported that OXPHOS and glycolysis metabolic pathways were required for protection against pathogenic microorganisms and that both were crucial for neutrophil homeostasis, migration, and inflammatory cytokine secretion [48]. Wang and McLean reported that M2 macrophages, regulatory T cells (Tregs), and memory T cells rely on OXPHOS and fatty acid oxidation [49]. Here, we provide clear evidence of links between the host, *Salmonella*, microbiota, and associated metabolites. The OXPHOS metabolic pathway and *MAVS* could be of interest for future study regarding the host, *Salmonella* and *Bacteroidetes*-derived propionate interactions.

Based on the correlation degree and the genes involved, four interesting modules were selected to determine the hub genes, namely the blue, brown, green, and yellow modules. In the blue module, genes such as *NDUFAF8*, *CDC27*, and *GOLGA4* were identified as hub genes, while only *MAVS* was identified in the brown. In contrast to the blue and brown modules, the green and yellow modules were negatively correlated to most traits. Notably, the green and yellow modules, were positively correlated with *Proteobacteria* cecal relative abundance. Genes such as *TADA2A* and *IL-1β* were identified as hub genes in the green module, while *C2CD5* and *ENSGALG00000052684* were identified as hub genes in the yellow module. The biological process analysis of these modules revealed that the brown and green modules displayed significantly enriched immune-related GO and KEGG pathways compared to the blue and yellow modules. However, the OXPHOS metabolic pathway was significantly enriched by the cluster of genes from the blue module. Furthermore, it was remarkable that the green module showed important immune-related GO and KEGG pathways, indicating that the genes contained in this module could be potential candidate genes. In addition, the green module was significantly and positively correlated with *Proteobacteria* relative abundance. These results suggest that *Proteobacteria* could play a crucial role in acquiring and developing adaptative immunity. The *Proteobacteria* relative abundance was significantly associated with genes involved in major immune-related biological processes such as leukocyte and T cell activation. Suggesting a potential involvement of this phylum in the activation and maturation of the immune system. Driver genes such as *FAM168B*, *USP8*, *C2CD5*, *PIGC*, *RAF1*, *EMC6*, *NFU1*, and several others were found shared among major factors involved in the resistance to *Salmonella* and could be potential candidate genes.

In this study, we also detected that the extracellular fatty acid binding protein (*EXFABP*) was significantly downregulated at 21 dpi compared to 7 dpi in chickens with low H/L ratio. In their study, Hu et al. demonstrated that *Salmonella* Enteritidis (OTU607) was positively correlated with *EXFABP* among others genes, indicating that *Salmonella* Enteritidis infection arouses *EXFABP* transcription in chicken, which induces the sequestration of siderophore secreted by enteric bacteria and Gram-positive bacilli, such as *Escherichia-Shigella* and *Enterococcus*, resulting in a decrease of their abundance [50]. During infection, *Salmonella* escapes the antibacterial effect of *EXFABP*-mediated growth inhibition through salmochelin, which is not recognized by *EXFABP* [50]. Thus, the inflammatory response induced by *Salmonella* increases *EXFABP* proteins and could limit the growth of *Enterobacteriaceae* [50]. The *Enterobacteriaceae* have a protective role by competing for oxygen and niche with *Salmonella* [51]. This could be one of the mechanisms of *Salmonella* for establishing successful infection. It is also possible that *EXFABP*-mediated inhibition of enteric bacteria could affect *Salmonella* despite salmochelin’s presence. To our knowledge, there is limited information on the enteric inhibitory growth effect of *EXFABP* on *Salmonella*. It was noteworthy that genes such as *EXFABP* and *S100A12* among others identified (Supplementary Table S2 GO and KEGG, L7 vs. L21 and H7 vs. H21) were involved in the enrichment of Gene Ontology (GO) terms: response to external stimuli (GO:0009605), defense response (GO:0006952), and extracellular region (GO:0005576). These results strongly suggest the potential involvement of *EXFABP* and *S100A12* in the host’s immune response and inhibition of *Salmonella* growth.

We further detected the expression of eight selected candidate genes in the cecum tissues of Dagu chickens infected with *Salmonella* Typhimurium, including *EMC6*, *FKBP5*, *S100A12*, *FAM168B*, *HESX1*, *NFU1*, and *PIGC*. We found that the expression of most genes changed significantly due to *Salmonella* infection, which indicates that these genes may participate in the host’s immune response to *Salmonella*. In this study, we found that *FKBP5* and *S100A12* were differentially expressed in chickens with high and low H/L ratios and between non-infected and ST-infected chickens. Moreover, at 7 dpi, the expression of *FKBP5* and *S100A12* were significantly upregulated in chickens with low H/L, which is consistent with their potential protective and immune enhancer functions reported in the literature.

In addition, we observed that the expression of cell migration inducing hyaluronan binding protein (*CEMIP*) was upregulated in chickens with low H/L ratios, compared to chickens with high H/L ratios at 21 dpi. Accordingly, Cazals and collaborators reported that *CEMIP*, among other genes, was significantly upregulated in both the resistant line N (low carriage) and in low carriers of the susceptible line (line 6) [52]. These results were in line with our findings indicating that chickens with low H/L ratios were more resistant than chickens with high H/L ratios at 21 dpi, and the *CEMIP* gene could be involved in the acquisition of resistance against *Salmonella* and maybe other pathogenic infections in other species. A study recently published by Dokoshi et al. reported that *CEMIP* regulates host defense against *Staphylococcus aureus* skin infection [53]. They found that *CEMIP* loss increases inflammation and antimicrobial activity following a skin infection and that CEMIP^−/−^ mice challenged with *S. aureus* had higher IL-6 and neutrophil infiltration [53]. These results indicate that *CEMIP* regulates inflammation and antimicrobial activity [53]. To date, the regulatory mechanisms of intestinal inflammation by *CEMIP* remain unelucidated. This is of further interest to understand the mechanisms of resistance to pathogenic intestinal infections.

Taken together, it is possible to suggest that at the earlier stage of infection, an overexpression of genes such as *FKBP5* and *S100A9/12* could confer enhanced immune response, while an overexpression of a gene such as *CEMIP* and the enrichment of OXPHOS pathway will exert an anti-inflammatory effect and antimicrobial activity. The strong immune response at the earlier stage of the infection will induce pathogen clearance and at the later stage post-infection, the regulation of the inflammation and enhanced antimicrobial activity will restore the intestinal homeostasis. In the present work, we identified the PPAR and OXPHOS metabolic pathways as involved in the mechanisms of resistance to *Salmonella* infection in chickens. The hub and driver genes detected in this study could contribute to developing new targets for control of *Salmonella*.

## 4. Materials and Methods

### 4.1. Animal, Experimental Design, and Sample Collection

A group of 200 one-day-old Jinxing yellow chicks from our previous study was used in the present work [17]. The birds were housed in sterilized isolation ventilated cages. Throughout the trial, the chicks received ad libitum Specific Pathogen Free (SPF) feed (Beijing Keao Xieli Feed Co., Ltd., Beijing, China) and free access to sterilized water. Before infection, all chicks were tested for *Salmonella* by culturing cloacal swab samples overnight at 37 °C with agitation in buffered peptone water [54]. No contaminated chicks were discovered, according to the results. At 7 days old, *Salmonella* Enteritidis 50335 (Institute of Veterinary drugs Control, Beijing, China) was used to challenge the birds with 1 mL of PBS containing 1 × 10^10^ CFUs of SE /mL.

The samples collection was performed at 7 and 21 days post-infection by randomly selecting 30 chickens. Before slaughter, the chicks were individually weighed and blood samples (1.5 mL distributed in one blood vial EDTA tube) were collected from the wings and stored at −20 °C. Next, the two ceca were aseptically sampled (section performed 2 cm from the junction ileocecal). After sectioning the ceca, sterile tweezers were used to squeeze the contents into sterile cryovial tubes for SCFAs and DNA extraction for 16S sequencing analysis. Then, the tissues were washed with PBS and stored in cryovial tubes at −80 °C for later DNA and RNA extraction.

### 4.2. Phenotype and Microbiome Relative Abundance Determination

The H/L ratios were determined using 10 µL of fresh blood, based on a method described elsewhere [19,55]. In brief, the blood smears were stained using Wright-Giemsa solution (G1020, Solarbio, Beijing, China) according to the manufacturer’s instructions. The concentration of propionate and valerate contents were measured by Gas Chromatography–Mass Spectrometry (GC-MS) using 100 mg of accurately weighted cecal contents [17]. The genomic DNA (gDNA) utilized in the current study was purified using a modified phenol–chloroform method. To quantify total *Salmonella* load in the cecum tissues, we used a method previously described elsewhere [16].

The cecal microbiota diversity was determined by 16S rRNA gene sequencing analysis [56], according to a method described elsewhere [17].

### 4.3. RNA Isolation

To extract the RNA from the cecum tissues, 32 cryopreserved samples were used (including 8 low and 8 high H/L ratios chickens from 7 and 21 dpi). Total RNA was isolated using the QIAGEN RNeasy Kit, and genomic DNA was removed using the TIANGEN DNase KIT (Tiagen, Beijing, China). The purity of the RNA was assessed using a kaiao K5500^®^ Spectrophotometer (Kaiao, Beijing, China), while the integrity and concentration of the RNA were determined using the RNA Nano 6000 Assay Kit and the Bioanalyzer 2100 system (Agilent Technologies, Santa Clara, CA, USA). A total of 2 µg of RNA were used as input material to synthesize the transcriptome analysis RNA samples. A total of 22 samples were utilized for transcriptome profiling based on the quality and purity of the isolated RNA. Cecal tissues from 4 to 7 individuals per group were used for transcriptome profiling and detection of genes differentially expressed.

### 4.4. Transcriptome Profiling and Differentially Expressed Genes

To understand the cecum’s gene transcription between high and low H/L ratio SE-infected chickens at the earlier and later stages post-infection, transcriptome data of 22 individuals were used (Supplementary Table S1). After filtering and quality control, more than 40 million clean reads were obtained. From the alignment of clean reads to the chicken reference genome (GRCg6a), a total of 22,701 genes were detected, with an average rate of 92.40% among all cecum samples. The transcriptome data were aligned in paired-end mode to the chicken reference genome (Ensembl GRCg6a) using the HISAT2 Version: 2.2.0 (https://daehwankimlab.github.io/hisat2/, accessed on 26 May 2021) with default settings. The NEBNext^®^ UltraTM RNA Library Prep Kit for Illumina^®^ (E7530L, New England Biolabs, Ipswich, MA, USA) was used to construct the sequencing libraries according to the manufacturer’s instructions, and index codes were applied to assign sequences to each sample. Purification of mRNA from the whole RNA was performed using poly-T oligo-attached magnetic beads. Next, fragmentation was carried out at elevated temperatures in the NEBNext First Strand Synthesis Reaction Buffer utilizing divalent cations (5X). The first strand of cDNA was produced with a random hexamer primer and RNase H, whereas the second strand was created with buffer, dNTPs, DNA polymerase I, and RNase H. Purification of library fragments was accomplished using QiaQuick PCR kits, followed by elution with EB buffer, terminal repair, A-tailing, and adaptor insertion. Finally, the target products were determined, the PCR reactions were performed, and the library was completed. The sequencing data were then subjected to quality control using FastQC (version 0.11.5) [57].

The differentially expressed genes (DEGs) were identified using DESeq2 [58] (Version 18.2.0) in the R programming language. The Wald test was used to determine the *p*-values, which were then adjusted using the Benjamini–Hochberg (BH) method [59]. The significance was set at a fold change of |log2 fold change| ≥ 1 and *padj* < 0.05.

### 4.5. Dynamic Developmental Genes Identification and Genes Expression Pattern

The normalized gene expression data of all constructed libraries were used to detect the developmental dynamics genes (DDGs). The DDGs were found using the maSigPro package (version 1.46.0) [60,61], which applied a negative binomial model to the expression distribution and adjusted the false discovery rate using the Benjamini and Hochberg approach. The “Backward” approach was used to pick significant genes with alpha equal to 0.05. The examination of gene expression patterns was conducted following the design of a single series time course. The following settings were used to cluster gene patterns: counts = TRUE, min.obs = 19, and rsq = 0.6. The clusters for data portioning (k) function with k.mclust = TRUE were utilized to obtain the optimal number of clusters.

### 4.6. Gene Ontology (GO) and Kyoto Encyclopedia of Genes and Genomes (KEGG) Pathway Analyses

To investigate the function of DEGs between the different H/L groups at 7 and 21 dpi, the ClusterProfiler package version 3.14 [62] and the org.Gg.eg.db package (version 3.14) [63] in R software was used to perform GO and KEGG pathway enrichment analyses. Based on the DEGs obtained from the comparative analysis of low and high H/L ratio groups from each dpi, GO and KEGG enrichment analyses were performed with a *p*-value of 0.05 stated as a threshold for significant enrichment.

### 4.7. Weighted Gene Co-Expression Network Analysis

A weighted gene co-expression network analysis was performed on all samples’ normalized gene expression data using the WGCNA (version 1.41) package [64] in R software, with some minor modifications. Out of the 22 samples from the transcriptome analysis, 19 were used to perform the weighted gene co-expression network analysis. The Fragments Per Kilobase per Million (FPKM) was utilized as a standardized measurement of transcription abundance to construct a gene expression matrix with a total of 19 samples, including 10 and 9 samples from 7 and 21 dpi, respectively (7 dpi: 4 low H/L ratio and 6 high H/L ratio chickens; 21 dpi: 4 low H/L ratio and 5 high H/L ratio chickens). The WGCNA default function removed the genes with low expression. The topological overlap matrix (TOM) was constructed using the step-by-step network construction method (soft-threshold equal to 8), with a minimum module size of 30 for the module detection. Next, we generated a cluster dendrogram including the module colors and merged it dynamically. The modules’ colors were merged at 0.25. The cluster dendrogram of co-expression network modules was generated using hierarchical clustering of genes based on the 1−TOM matrix. To assess associations of co-expressed gene clusters with dpi, the 7 and 21 dpi groups were assigned nominal values of 1 and 2, respectively; for the gene clusters with H/L group, the low and high H/L ratio chicken groups were assigned nominal values of 0 and 1, respectively [65,66,67]. The association of co-expressed genes with the other traits was also evaluated. High absolute values of gene significance (|GS| > 0.5) and module membership (|MM| > 0.5) with a threshold of *p*-value < 0.01 were used to identify the driver genes [65,68]. Gene co-expression networks were determined using Cytoscape version 3.6.0 [69] with the edges and nodes provided by the WGCNA “exportNetworkToCytoscape” function. Next, the genes with a high weight based on the intramodular connectivity were identified as hub genes [70,71].

### 4.8. Quantitative Real-Time PCR

Dagu chickens were orally infected by *Salmonella* Typhimurium 21484 (ST, China Industrial Microbial Culture Preservation Center, Beijing, China) with 1 mL of PBS containing 1.5 × 10^13^ CFU of ST/mL at 14 days old. The chicks from the non-infected group were given the same volume of sterile PBS. In this work, the cecum of 6 chickens in the infection group and 6 chickens in the control group were randomly selected to extract RNA to verify the expression of candidate genes. The total RNA of the cecum was extracted by Trizol reagent (Invitrogen). RNA (1000 ng) was reverse transcribed by cDNA synthesis kit (TIANGEN) for quantitative real-time PCR. Primers were designed according to chicken coding region sequences and synthesized by The Beijing Genomics Institute (BGI), listed in Supplementary Table S7. Data were normalized to the expression of the housekeeping gene *ACTB*. Quantitative real-time PCR was performed in triplicate using the Invitrogen PowerUp™ SYBR^®^ Green Master Mix (ABI) with the following cycle profile: 95 °C for 3 min, 40 cycles of 95 °C for 3 s, and annealing temperature for 34 s in the QuantStuio 7 Flex Real-Time PCR System (Waltham, MA, USA). The results were analyzed by 2^−∆∆Ct^ method [72].

### 4.9. Statistical Analysis

The data were analyzed using GraphPad Prism version 8 (GraphPad Software, San Diego, CA, USA) and R version 4.1. Two-way ANOVA with Sidak’s multiple comparisons analyzed differences between low and high H/L ratio groups at 7 and 21 dpi. Kruskal–Wallis’s sum rank test analyzed the four groups and detected significant differential abundance features at the phylum level. Assessment of DEGs, DDGs, and driver genes shared between different groups was performed using jvenn, an open-source online tool for comparing lists using Venn Diagrams (http://bioinfo.genotoul.fr/jvenn, accessed on 26 May 2021) [73]. The results are expressed as the mean and standard error of the mean (SEM). All significance was declared when *p* < 0.05.

## Figures and Tables

**Table 1:** Top DEGs.

Groups	Ensemble ID	Gene Symbol	Log2FC	pval	padj.
**H7 vs. L7**	ENSGALG00000043754	*GLUL*	−1.15	2.24E−06	0.0171
	ENSGALG00000038540	*ZBTB16*	−1.88	1.46E−05	0.0346
	ENSGALG00000041202	*FBXO32*	−1.19	1.32E−05	0.0346
	ENSGALG00000042148	*FKBP5*	−1.87	1.89E−05	0.0359
	ENSGALG00000009700	*PDK4*	−1.34	3.55E−05	0.0490
	ENSGALG00000016448	*KCNF1*	−1.04	3.27E−05	0.0490
	ENSGALG00000044940	*GCNT3*	1.01	0.0002	0.1347
**H21 vs. L21**	ENSGALG00000029006	*SYPL1*	1.55	9.26E−06	0.1911
	ENSGALG00000041921	*CWH43*	1.40	5.39E−05	0.5567
	ENSGALG00000002605	*MRPL17*	1.04	0.0008	0.9946
	ENSGALG00000020827	*ATP13A4*	4.17	0.0015	0.9946
	ENSGALG00000030502	*ADAMTS4*	−1.65	0.0018	0.9946
	ENSGALG00000009919	*KCNG3*	1.38	0.0018	0.9946
	ENSGALG00000006413	*CEMIP*	−1.27	0.0018	0.9946
	ENSGALG00000036659	*NDUFAF8*	1.27	0.0025	0.9946
**H7 vs. H21**	ENSGALG00000026098	*IL8*	2.46	2.19E−19	1.67E−15
	ENSGALG00000003876	*TIMD4*	−1.67	3.00E−17	1.44E−13
	ENSGALG00000015307	*-*	−1.32	2.22E−15	6.78E−12
	ENSGALG00000027786	*SOCS3*	1.99	2.90E−15	7.38E−12
	ENSGALG00000016186	*PDE9A*	1.59	6.76E−14	1.47E−10
	ENSGALG00000015733	*MDN1*	−1.01	7.97E−14	1.52E−10
	ENSGALG00000001252	*CREB3L3*	3.45	9.26E−13	1.57E−09
	ENSGALG00000008518	*-*	1.13	1.79E−12	2.49E−09
	ENSGALG00000010961	*IGF2BP3*	1.18	1.79E−12	2.49E−09
	ENSGALG00000005521	*PER2*	−1.08	2.13E−12	2.72E−09
**L7 vs. L21**	ENSGALG00000043064	*EXFABP*	4.23	7.98E−16	1.21E−11
	ENSGALG00000004228	*USP40*	−1.62	9.62E−15	6.00E−11
	ENSGALG00000025945	*AVD*	5.60	1.19E−14	6.00E−11
	ENSGALG00000033807	*TYSND1*	1.19	6.23E−12	1.05E−08
	ENSGALG00000002722	*MST1*	2.03	1.30E−11	1.64E−08
	ENSGALG00000053916	*ABCB1*	−1.22	2.74E−10	2.18E−07
	ENSGALG00000033204	*PQLC2*	1.00	5.27E−10	3.99E−07
	ENSGALG00000024272	*S100A9*	5.69	8.29E−10	5.71E−07
	ENSGALG00000041403	*DENND4A*	−1.02	1.82E−09	1.20E−06

Note: Log2FC refers to Log2 fold change.

**Table 2:** Top ten driver genes in the significant modules.

Trait	Correlation *	Module Color	Gene Names (GS, MM)
**Dpi**	Positive	Blue	*PER2 (0.95, 0.86), ROCK2 (0.94, 0.84), USP8 (0.94, 0.83), GOLGA4 (0.93, 0.87), CDC42SE1 (−0.93, −0.86), ENSGALG00000029691 (−0.93, −0.80), PIGC (−0.91, −0.83), CKAP2L (−0.91, −0.78), H3F3B (−0.91, −0.79), C8H1orf52 (−0.90, −0.77)*
		Brown	*CGNL1 (0.87, 0.77), TLN2 (0.85, 0.79), HRH1 (0.85, 0.77), ECM2 (0.85, 0.87), TMEM245 (0.84, 0.66), NFU1 (−0.91, −0.70), EMC6 (−0.88, −0.70), ENSGALG00000021686 (−0.81, −0.84), GNG5 (−0.81, −0.81), JAGN1 (−0.80, −0.72)*
	Negative	Yellow	*FAM168B (0.89, −0.83), C2CD5 (0.88, −0.86), RAF1 (0.87, −0.78), HESX1 (0.83, −0.70), LYPLA1 (0.80, −0.83), DDTL (0.79, −0.74), ENSGALG00000053041 (−0.91, 0.87), ENSGALG00000043126 (−0.90, 0.82), MARCKSL1 (−0.89, 0.83), TMEM115 (−0.89, 0.84)*
**H/L ratio**	Positive	Dark-green	*ENSGALG00000038918 (0.62, 0.66), DNAJC17 (0.65, 0.52)*
**Body weight post-** **infection**	Positive	Blue	*GOLGA4 (0.92, 0.87), ENSGALG00000048303 (0.91, 0.74), ROCK2 (0.90, 0.84), USP8 (0.90, 0.83), PER2 (0.90, 0.86), ORMDL2 (−0.92, −0.86), CDC42SE1 (−0.92, −0.86), UBALD1 (−0.91, −0.91), ENSGALG00000029691 (−0.90, −0.80), PIGC (−0.90, −0.83)*
	Negative	Yellow	*C2CD5 (0.89, −0.86), RAF1 (0.87, −0.78), DDTL (0.84, −0.74), FAM168B (0.84, −0.83), PLA2G4F (0.82, −0.89), HESX1 (0.82, −0.70), ENSGALG00000053041 (−0.90, 0.87), TMEM115 (−0.89, 0.84), UBE2M (−0.89, 0.90), ENSGALG00000052202 (−0.89, 0.91)*
**Bacterial load (SE)**	Positive	Dark-turquoise	*SOWAHA (0.73, 0.70), ENSGALG00000007596 (0.73, 0.67), ENSGALG00000040718 (0.72, 0.69), STBD1 (0.71, 0.75), HMX3 (0.70, 0.57), ENSGALG00000050785 (0.58, 0.68), ENSGALG00000050054 (0.58, 0.80), RASSF10 (0.59, 0.70), ENSGALG00000049917 (0.59, 0.92), LYPD6 (0.60, 0.74)*
		Magenta	*CREB3L3 (0.82, 0.69), ENSGALG00000045581 (0.81, 0.74), ENSGALG00000029381 (0.80, 0.78), WFDC2 (0.80, 0.73), ENSGALG00000007645 (0.78, 0.84), GXYLT2 (−0.63, −0.74), GTF2E2 (−0.59, −0.72), EEF1A2 (0.58, 0.83), RNF128 (0.58, 0.93), MLN (0.58, 0.97)*
	Negative	Blue	*TMEM243 (0.89, −0.74), ENSGALG00000049966 (0.87, −0.83), FOXA1 (0.87, −0.80), OTP (0.86, −0.60), SDF2 (0.85, −0.93), PI4KA (−0.86, 0.87), INPP5K (−0.83, 0.87), ENSGALG00000004881 (−0.83, 0.91), TUBGCP4 (−0.83, 0.79), ENSGALG00000053997 (−0.83, 0.92)*
		Pink	*FUCA1 (0.86, −0.69), ENSGALG00000051392 (0.78, −0.69), ENSGALG00000047357 (0.67, −0.77), ENSA (0.65, −0.86), IRF2 (0.65, −0.78), NABP1 (−0.77, 0.80), NVL (−0.76, 0.79), LARS1 (−0.75, 0.86), NRDC (−0.74, 0.89), MKLN1 (−0.73, 0.84)*
		Brown	*ENSGALG00000047515 (0.75, −0.66), EFNB1 (0.75, −0.76), ENSGALG00000048205 (0.73, −0.74), ENSGALG00000021686 (0.73, −0.84), ENSGALG00000048159 (0.72, −0.69), NFU1 (0.71, −0.70), EMC6 (0.69, −0.70), PCMT1 (−0.81, 0.77), TMEM245 (−0.77, 0.66), CD109 (−0.73, 0.83)*
**Propionate**	Positive	Blue	*PER2 (0.86, 0.86), USP8 (0.86, 0.83), SLC25A1 (0.86, 0.71), NEK1 (0.85, 0.88), TIMD4 (0.85, 0.84), C8H1orf52 (−0.79, −0.77), PIGC (−0.79, −0.83), CKAP2L (−0.79, −0.78), H3F3B (−0.79, −0.79), ENSGALG00000029691 (−0.79, −0.80)*
		Tan	*HECW1 (0.81, 0.74), LGI2 (0.77, 0.70), ENSGALG00000035854 (0.75, 0.85), SLC6A15 (0.74, 0.56), TACSTD2 (0.72, 0.69), ENSGALG00000054224 (0.72, 0.80), ENSGALG00000044674 (0.58, 0.96), ENSGALG00000013762 (0.58, 0.80), CNDP1 (0.58, 0.77), NTNG1 (0.59, 0.76)*
		Brown	*TLN2 (0.87, 0.79), ECM2 (0.86, 0.87), HRH1 (0.81, 0.77), CGNL1 (0.81, 0.77), ENSGALG00000048205 (−0.79, −0.74), EMC6 (−0.78, −0.70), ENSGALG00000021686 (−0.78, −0.84), NFU1 (−0.76, −0.70), JAGN1 (−0.71, −0.72), GNG5 (−0.71, −0.81)*
	Negative	Yellow	*C2CD5 (0.80, −0.86), HESX1 (0.79, −0.70), ENSGALG00000017139 (0.78, −0.80), FAM168B (0.78, −0.83), SASH1 (0.78, −0.61), ENSGALG00000004725 (0.75, −0.75), RAF1 (0.75, −0.78), CYP2U1 (0.74, −0.75), ENSGALG00000043126 (−0.82, 0.82), ENSGALG00000053041 (−0.81, 0.87)*
**Valerate**	Positive	Blue	*TIMD4 (0.91, 0.84), NEK1 (091, 0.88), USP8 (0.86, 0.83), ROCK2 (0.85, 0.84), ORMDL2 (−0.88, −0.86), CKAP2L (−0.87, −0.78), C8H1orf52 (−0.86, −0.77), PIGC (−0.85, −0.83), CDC42SE1 (−0.83, −0.86), H3F3B (−0.82, −0.79)*
		Tan	*ENSGALG00000035854 (0.74, 0.85), NT5E (0.72, 0.80), LGI2 (0.71, 0.70), ENSGALG00000052840 (0.70, 0.65), CCDC148 (0.69, 0.61), SLC16A12 (0.67, 0.67), ASIP (0.58, 0.85), ITGA11 (0.58, 0.63), KCNV1 (0.59, 0.53), ENSGALG00000054224 (0.59, 0.80)*
		Brown	*CGNL1 (0.90, 0.77), TLN2 (0.87, 0.79), HRH1 (0.85, 0.77), ECM2 (0.80, 0.87), ENSGALG00000021686 (−0.80, −0.84), NFU1 (−0.79, −0.70), EMC6 (−0.76, −0.70), ENSGALG00000048205 (−0.74, −0.74), GNG5 (−0.72, −0.81), JAGN1 (−0.68, −0.72)*
	Negative	Yellow	*C2CD5 (0.82, −0.86), HESX1 (0.78, −0.70), FAM168B (0.77, −0.83), RAF1 (0.75, −0.78), DDTL (0.75, −0.74), CYP2U1 (0.73, −0.75), ENSGALG00000053041 (−0.84, 0.87), ENSGALG00000043126 (−0.84, 0.82), UBE2M (−0.83, 0.90), TMEM115 (−0.81, 0.84)*
***Firmicutes***	Positive	Gray	*CERS5 (0.56, 0.59), FKBP3 (−0.53, −0.76), HPF1 (−0.56, −0.63)*
	Negative	Blue	*CALML4 (0.77, −0.57), H-RAS (0.77, −0.83), ENSGALG00000046988 (0.74, −0.59), ACSL5 (0.73, −0.61), ENSGALG00000004503 (0.71, −0.69), CNGA3 (−0.77, 0.65), ENSGALG00000001972 (−0.75, 0.80), DOCK7 (−0.75, 0.61), DENND5B (−0.73, 0.63), ADCY2 (−0.73, 0.71)*
		Pink	*EHHADH (0.78, −0.73), AADAC (0.67, −0.74), FAM81A (0.66, −0.58), ZDHHC9 (0.65, −0.80), IRF2 (0.64, −0.78), GPR176 (−0.72, 0.53), GAR1 (−0.72, 0.72), URI1 (−0.70, 0.82), CXCR4 (−0.69, 0.73), SRSF2 (−0.69, 0.72)*
***Bacteroidetes***	Positive	Blue	*SLC25A16 (0.86, 0.71), BAG3 (0.88, 0.63), ENSGALG00000048303 (0.85, 0.74), MYBL1 (0.85, 0.69), CDC27 (0.84, 0.78), ORMDL2 (−0.83, −0.86), H-RAS (−0.83, −0.83), GPX1 (−0.80, −0.74), PIGC (−0.80, −0.83), NHEJ1 (−0.79, −0.82)*
		Pink	*ENSGALG00000045350 (0.76, 0.74), DNAJA2 (0.70, 0.73), FAM98A (0.70, 0.80), NEIL3 (0.70, 0.74), MKLN1 (0.69, 0.84), FUCA1 (−0.60, −0.69), ENSA (−0.58, −0.86), SDAD1 (0.58, 0.91), CCT6A (0.58, 0.87), SIKE1 (0.58, 0.82)*
		Tan	*ENSGALG00000035854 (0.79, 0.85), MTURN (0.71, 0.78), RPP14 (0.69, 0.82), ENSGALG00000005938 (0.58, 0.87), PCDH10 (0.59, 0.62), FKBP1B (0.59, 0.62), PDCL2 (0.59, 0.63), SULT (0.60, 0.71), ENSGALG00000051072 (0.60, 0.67)*
	Negative	Yellow	*RAF1 (0.76, −0.78), HESX1 (0.73, −0.70), IKBKB (0.73, −0.77), FAM168B (0.72, −0.83), LYPLA1 (0.70, −0.83), PLA2G4F (0.70, −0.89), ENSGALG00000052202 (−0.79, 0.91), MARCKSL1 (−0.77, 0.83), ZNF142 (−0.85, 0.84), XKR8 (−0.84, 0.79)*
***Proteobacteria***	Positive	Black	*ENSGALG00000052317 (0.89, 0.73), AMIGO3 (0.87, 0.86), RFT1 (0.87, 0.74), ENSGALG00000035539 (0.87, 0.72), MEPE (0.86, 0.87), UQCRQ (−0.68, −0.52), ANAPC5 (−0.63, −0.58)*
		Green	*WNT5B (0.85, 0.85), SUN2 (0.83, 0.80), PCNX2 (0.82, 0.91), ITGA2B (0.80, 0.80), OLFM4 (0.80, 0.80), UGT1A1 9−0.76, −0.68), SFRP1 (−0.74, −0.56), ACAA1 (−0.66, −0.55), COMTD1 (−0.65, −0.60), PDLIM1 (−0.61, −0.65)*
		Yellow	*JPH3 (0.91, 0.81), CCDC130 (0.90, 0.82), TAF6 (0.85, 0.85), ATP13A2 (0.84, 0.86), C2CD4C (0.83, 0.76), EPB41L3 (−0.75, −0.86), OCIAD1 (−0.69, −0.65), TSKU (−0.69, −0.73), WBP2 (−0.69, −0.71), ANK3 (−0.67, −0.92)*

Note: GS, Gene Significance; MM, Module Membership. * Correlation between the trait and the module.

## Data Availability

The transcriptome data presented in this study are available in the Genome Sequence Archive repository (https://ngdc.cncb.ac.cn/gsa/, accessed on 13 February 2023), accession number CRA009860.

## Supplementary Materials

The following supporting information can be downloaded at: https://www.mdpi.com/article/10.3390/ijms24054824/s1.
