# Supplementary material for: Gene Co-Expression Network Analysis Reveals the Hub Genes and Key Pathways Associated with Resistance to Salmonella Enteritidis Colonization in Chicken
Source: Int J Mol Sci. 2023 Mar 2;24(5):4824. doi: 10.3390/ijms24054824 (PMC10003191; doi:10.3390/ijms24054824)
Supplement: Supplementary file 1 [file ijms-24-04824-s001.zip › ijms-2061148-supplementary figures.pdf]

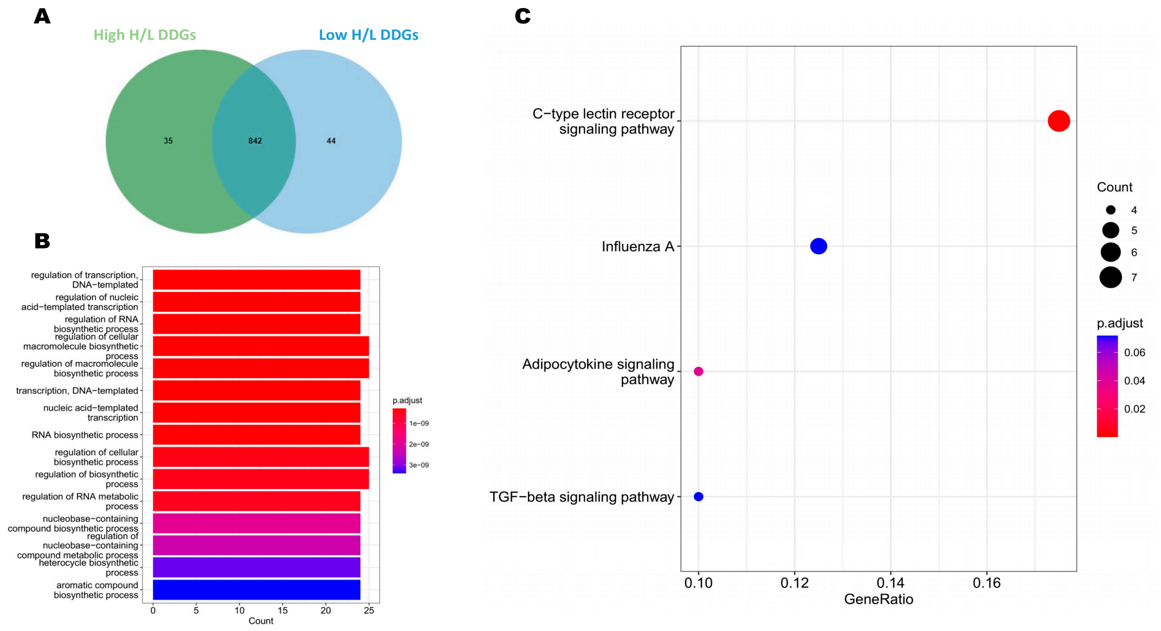

Supplementary Figure S1: Developmentally Dynamic Genes (DDGs), GO, and KEGG of the transcript factors (TFs) and co-factors of these DDGs. A) Overlap of DDGs between low and high H/L ratio group. B) Top 15 enriched GO terms for TFs of DDGs. C) KEGG pathways enrichment for TFs of DDGs.

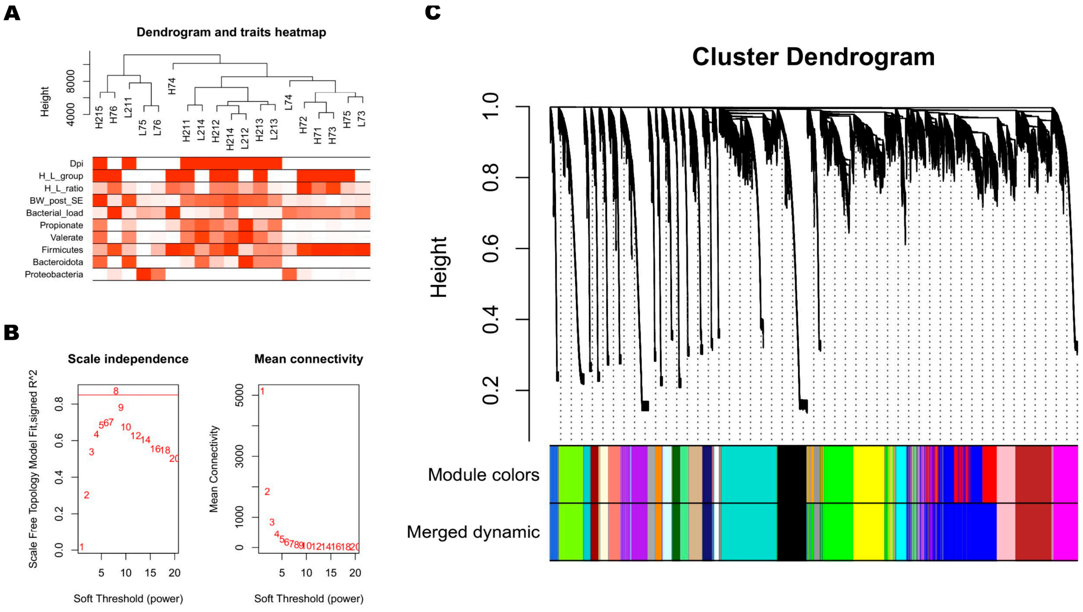

Supplementary Figure S2: Weighted Gene-Co-Expression Network Analysis (WGCNA) results show the modules significantly correlated with different immune-related traits. A) Dendrogram and traits heatmap. B) Scale-free topology model and Mean connectivity. C) Cluster dendrogram reveals the module's colors and the merged dynamic (n = 19).

Supplementary Table S1: Summary statistics of the RNA-seq data.
Supplementary Table S2: GO terms and KEGG pathways enrichment of transcriptome analysis.
Supplementary Table S3: list of Transcript Factors (TFs) and Transcription Co-Factors (TCFs).
Supplementary Table S4: GO terms and KEGG pathways of TFs and TCFs.
Supplementary Table S5: GO terms and KEEG pathways of the four interesting modules (Blue, Brown, Green and Yellow).
Supplementary Table S6: Unique and shared driver genes among different traits.
Supplementary Table S7: sequences of primers used for qRT-PCR verification.
